# Omics data reveal the unusual asexual-fruiting nature and secondary metabolic potentials of the medicinal fungus *Cordyceps cicadae*

**DOI:** 10.1186/s12864-017-4060-4

**Published:** 2017-08-30

**Authors:** Yuzhen Lu, Feifei Luo, Kai Cen, Guohua Xiao, Ying Yin, Chunru Li, Zengzhi Li, Shuai Zhan, Huizhan Zhang, Chengshu Wang

**Affiliations:** 1 CAS Key Laboratory of Insect Developmental and Evolutionary Biology, Shanghai Institute of Plant Physiology and Ecology, Chinese Academy of Sciences, Shanghai, 200032 China; 2 University of Chinese Academy of Sciences, Beijing, 100049 China; 3 School of Biotechnology, East China University of Science and Technology, Shanghai, 200237 China; 4 School of Computer Science, Fudan University, Shanghai, 200433 China; 5 Zhejiang BioAsia Institute of Life Science, Pinghu, 314000 China

**Keywords:** *Cordyceps cicadae*, Genomics, Mating type, Asexual fruiting, Secondary metabolism, Bioactive metabolites

## Abstract

**Background:**

Ascomycete *Cordyceps* species have been using as valued traditional Chinese medicines. Particularly, the fruiting bodies of *Cordyceps cicadae* (syn. *Isaria cicadae*) have long been utilized for the treatment of chronic kidney disease. However, the genetics and bioactive chemicals in this fungus have been largely unexplored.

**Results:**

In this study, we performed comprehensive omics analyses of *C. cicadae*, and found that, in contrast to other *Cordyceps* fungi, *C. cicadae* produces asexual fruiting bodies with the production of conidial spores instead of the meiotic ascospores. Genome sequencing and comparative genomic analysis indicate that the protein families encoded by *C. cicadae* are typical of entomopathogenic fungi, including the expansion of proteases and chitinases for targeting insect hosts. Interestingly, we found that the MAT1-2 mating-type locus of the sequenced strain contains an abnormally truncated *MAT1-1-1* gene. Gene deletions revealed that asexual fruiting of *C. cicadae* is independent of the MAT locus control. RNA-seq transcriptome data also indicate that, compared to growth in a liquid culture, the putative genes involved in mating and meiosis processes were not up-regulated during fungal fruiting, further supporting asexual reproduction in this fungus. The genome of *C. cicadae* encodes an array of conservative and divergent gene clusters for secondary metabolisms. Based on our analysis, the production of known carcinogenic metabolites by this fungus could be potentially precluded. However, the confirmed production of oosporein raises health concerns about the frequent consumption of fungal fruiting bodies.

**Conclusions:**

The results of this study expand our knowledge of fungal genetics that asexual fruiting can occur independent of the MAT locus control. The obtained genomic and metabolomic data will benefit future investigations of this fungus for medicinal uses.

**Electronic supplementary material:**

The online version of this article (doi:10.1186/s12864-017-4060-4) contains supplementary material, which is available to authorized users.

## Background

Ascomycete fungi belonging to *Cordyceps* sensu lato account to more than 500 known species that are classified into the three families, Cordycipitaceae, Ophiocordycipitaceae, and Clavicipitaceae [1, 2]. The family Cordycipitaceae includes the genera *Cordyceps*, *Isaria*, *Beauveria*, and *Lecanicillium*. The sexual stages of several species belonging to the genera *Beauveria* and *Lecanicillium* have been clarified as the *Cordyceps* spp. [1, 3]. In the family Ophiocordycipitaceae, some species such as *Ophiocordyceps sinensis* (better known as the caterpillar fungus *C. sinensis*) and *O. unilateralis* are highly host-specific and the infections by these fungi can alter insect host behaviors [4, 5]. The mechanisms of entomopathogenicity have been well studied in a number of species including *Beauveria* spp., and *Metarhizium* spp. belonging to the family Clavicipitaceae [2], which have been frequently using as insect biocontrol agents [6, 7]. The fruiting bodies produced by *C. militaris*, *O. sinensis*, and *C. cicadae* have long been used as valued traditional Chinese medicines (TCMs) for anticancer, immunomodulation, anti-fatigue, and anti-impotence [8, 9]. *C. cicadae* has once been called as *C. sobolifera*, *C. sinclairii*, *Paecilomyces cicadae*, and *Isaria cicadae*. The last name has become the recognized synonym of this fungus [10]. In contrast to *C. militaris* and *O. sinensis* with clear sexual lifecycles [11], the sexuality of *C. cicadae* is still enigmatic.

In *Cordyceps*, sexual reproduction is either heterothallic or homothallic that is controlled by the mating-type (MAT) loci [11]. The MAT1-1 locus in general contains two-three genes whereas the MAT1-2 locus has only one *MAT1-2-1* gene [11, 12]. For example, the MAT1-1 loci in *Metarhizium* species contain three genes (i.e., *MAT1-1-1*, *MAT1-1-2* and *MAT1-1-3*) whereas the MAT1-1 loci in *B. bassiana* and *C. militaris* have two genes [11, 13]. Interestingly, recent genome sequencing of different *B. bassiana* strains identified an additional *MAT1-2-8* gene in the MAT1-2 type isolates [14]. Loss-of-function studies of *MAT* genes in *C. militaris* have indicated that both MAT1-1 and MAT1-2 loci are required for fungal fruiting and fertility but the three genes *MAT1-1-1*, *MAT1-1-2*, and *MAT1-2-1* play diverse roles in regulating fertility and the formation of perithecia, asci, or ascospore [15]. In the homothallic fungus *Fusarium graminearum*, all *MAT* genes are not required for the formation of perithecia [16]. Until this study, the structure of MAT loci in *C. cicadae* is unclear, and their precise functions remain unknown.

In Asia, the fruiting bodies of *C. cicadae* together with the mycosed cicada cadaver are used as medicinal herbs for the treatment of chronic kidney disease [17, 18]. One study reported that the anti-fibrotic activity of ergosterol peroxide isolated from *C. cicadae* was responsible for its renoprotective effect [18]. Other studies exploring bioactive metabolites identified different adenosine analogs and cyclodepsipeptides such as cordycecin A and beauvericins in *C. cicadae* [19, 20]. Reports on whether or not *C. cicadae* can produce the anticancer compound cordycepin (i.e., 3′-deoxyadenosine) are inconsistent [21, 22]. The potent immunosuppressant drug Fingolimod was developed from the atypical amino acid myrocin produced by *Isaria sinclairii* [23]. Moreover, safety concerns of consuming *Cordyceps* have also been frequently raised due to uncertainty on fungal production of human-toxic mycotoxins [9, 24].

In this study, we performed fruiting-body induction, de novo genome sequencing and comparative genomic analysis of *C. cicadae* to help understand the genetic nature of fungal developmental controls. Fungal development-associated metabolomic analyses, transcriptomics, and deletion of *MAT* genes were also performed to better understand the secondary metabolisms and genetics of this fungus.

## Results

### Asexual fruiting and insect pathogenicity of *C. cicadae*

**Fig. 1:**
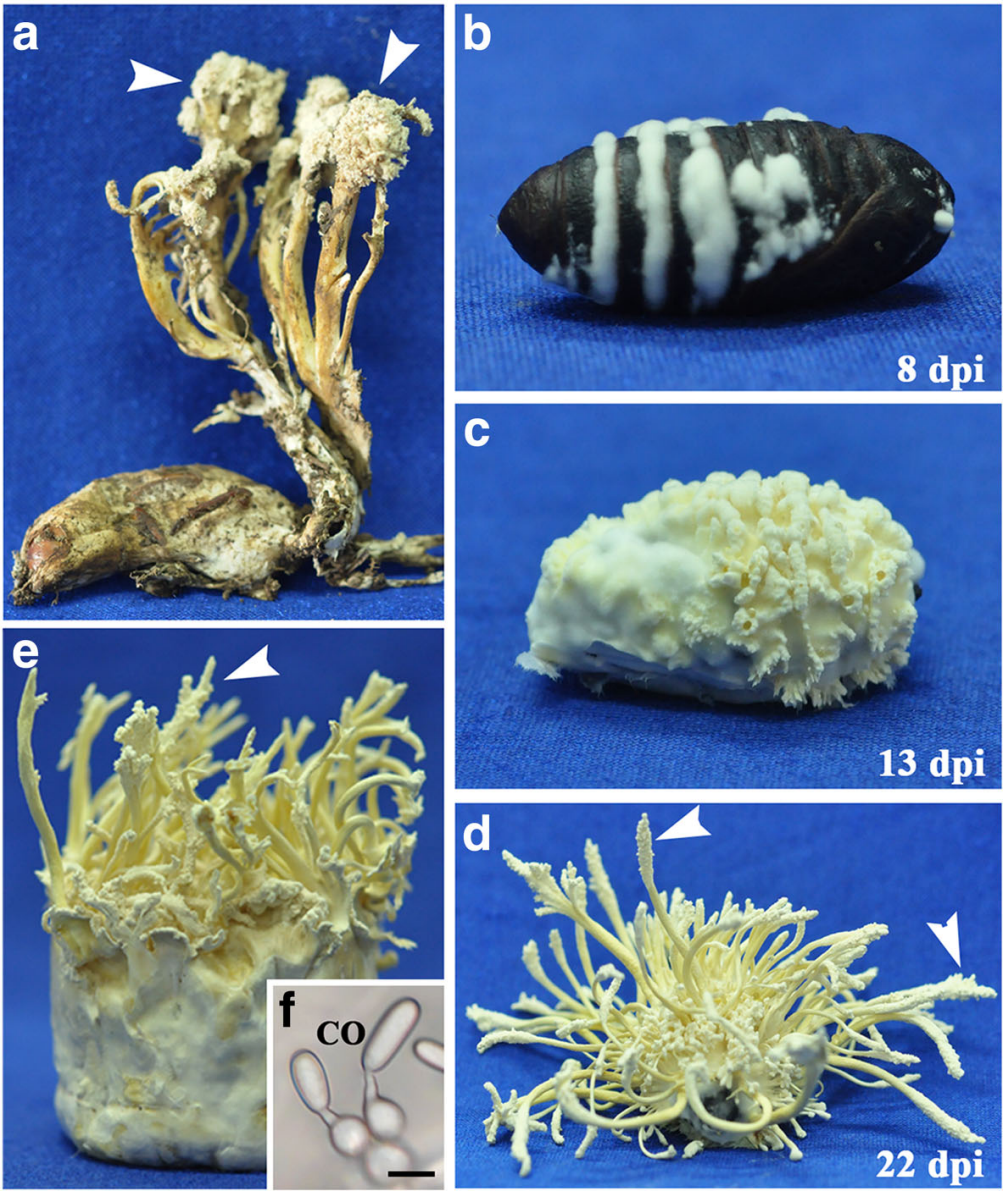
Phenotypes and development of asexual fruiting in *C. cicadae*. **a** Field collected fruiting bodies of *C. cicadae* developed on the cadaver of cicada *Platylomia* sp. **b**-**d** Fruiting body production of *C. cicadae* on the pupae of Chinese tussah silkworm (*Antheraea pernyi*) after inoculation for different times (labeled in each panel). Dpi, days post inoculation. **e** Fruiting bodies of *C. cicadae* produced on the rice medium 23 days post inoculation. **f** Asexual conidial spores produced on the fruiting bodies (arrows in the panels A, D and E). CO, conidium; Bar, 5 μm

In nature, the fruiting bodies of *C. cicadae* form uniquely on pupated cicadae (*Platylomia* spp.) (Fig. 1a) and are referred to as “cicada flower” in China [25]. Due to the difficulty in rearing cicadae in the laboratory, we used the pupae of Chinese tussah silkworm (*Antheraea pernyi*) for inoculation with the conidia of *C. cicadae*. We found that the silkworm pupae could be killed and mycosed by the fungus 8 days post inoculation (dpi) (Fig. 1b). Fungal primordia formed 13 dpi (Fig. 1c), and the mature stroma/fruiting bodies were produced 22 dpi (Fig. 1d). The stroma of *C. cicadae* formed on artificial rice medium ca. 3 weeks post inoculation (Fig. 1e). Microscopic examinations indicated that the asexual conidial spores but not the sexual ascospores were produced on field-collected or lab-induced fruiting bodies (Fig. 1f). Thus, in contrast to other *Cordyceps* species that produce sexual fruiting bodies in the fields [1], *C. cicadae* forms synnema-like asexual structures.

To examine the ability of *C. cicadae* to infect non-cicada insect hosts, we induced formation of the infection structure appressorium on the hind wings of the mealworm *Tenebrio molitor*. The results revealed that unlike *C. militaris*, *C. cicadae* could produce appressoria on beetle wings similar to *M. robertsii*, *B. bassiana*, and *I. fumosorosea* (Additional file 1: Fig. S1A). Insect bioassays against the mealworm larvae confirmed that *C. cicadae* could kill insects like *B. bassiana* and *M. robertsii* within 4 to 5 days post topical infection whereas *C. militaris* could not infect beetles (Additional file 1: Fig. S1B). In addition, we found that both *C. cicadae* and *I. fumosorosea* but not *M. robertsii* and *B. bassiana* could form synnema-like structures on insect cadavers (Additional file 1: Fig. S1C).

### Genome sequencing and protein families involved in fungal entomopathogenicity

To better understand the physiology of *C. cicadae*, de novo genome sequencing was performed to obtain a 98.8% completeness of the genome. The genome size (33.9 Mb) and gene-coding capacity (9701 genes) of the fungus are equivalent to four other ascomycete insect pathogenic fungi *C. militaris*, *I. fumosorosea*, *B. bassiana*, and *M. robertsii* (Table 1). Protein family analyses indicated that *C. cicadae* has fewer numbers of total conserved protein families, and putative proteins involved in pathogen-host interaction (PHI) when compared to other pathogens. However, *C. cicadae* encodes higher numbers of small secreted cysteine-rich proteins (SSCPs), proteases, G-protein coupled receptors (GPCRs), lipases, glycoside hydrolases (GHs), and core genes involved in secondary metabolisms when compared to *C. militaris* or other fungi (Table 1). For example, *C. cicadae* encodes more lipases (35) than *C. militaris* (23), *I. fumosorosea* (21), and *B. bassiana* (28). In addition, more bacterial-like protein toxins are encoded by *C. cicadae* (16) than by *C. militaris* (6) and *I. fumosorosea* (12) but fewer than by *B. bassiana* (26) (Table 1). Overall, *C. cicadae* has the conventional genome features of entomopathogenic fungi [2, 26], including the genome expansion of serine proteases and chitinases (GH18) that are used by the fungus to degrade the protein- and chitin-rich cuticles of insect hosts (Additional file 1: Table S1-S4).

**Table 1:** Comparison of the sequencing and genome features of *C. cicadae* with other entomopathogenic fungi

Features^a^	*C. cicadae*	*I. fumosorosea*	*C. militaris*	*B. bassiana*	*M. robertsii*
Size (Mb)	33.9	33.5	32.2	33.7	39
Coverage fold	80×	86.99×	147×	76.6 ×	100×
Scaffold no. (>1 kb)	599	430	13	242	176
Scaffold N50 (Mb)	0.21	0.87	4.55	0.73	1.96
% G + C content	53.0	53.6	51.4	51.5	51.5
% Repeat rate	3.19	3.84	3.04	2.03	0.98
Protein-coding genes	9701	10,060	9684	10,366	10,582
Gene density (per Mb)	286	300	301	308	271
Exons per gene	2.6	2.6	3	2.7	2.8
Protein families	2592	2876	2736	3002	2797
Putative PHI genes	1490	1604	1547	2121	1828
SSCPs	268	287	207	305	283
Secreted proteins	1031	1390	1133	1378	1333
Proteases	388	395	371	386	390
Secreted proteases	127	158	120	142	1660
GPCRs	38	39	29	32	64
Lipases	29	21	23	28	35
Secondary metabolisms	34	36	29	42	62
Glycoside hydrolases	135	143	134	144	149
Cytochrome P450s	67	76	65	89	133
Bacterial-like toxins	16	12	6	26	16

^a^,Abbreviations: *Mb* mega base, *PHI* pathogen-host interaction, *SSCP* small secreted cysteine-rich protein, *GPCR* G-protein coupled receptor

### Phylogenetic and syntenic relationships

**Fig. 2:**
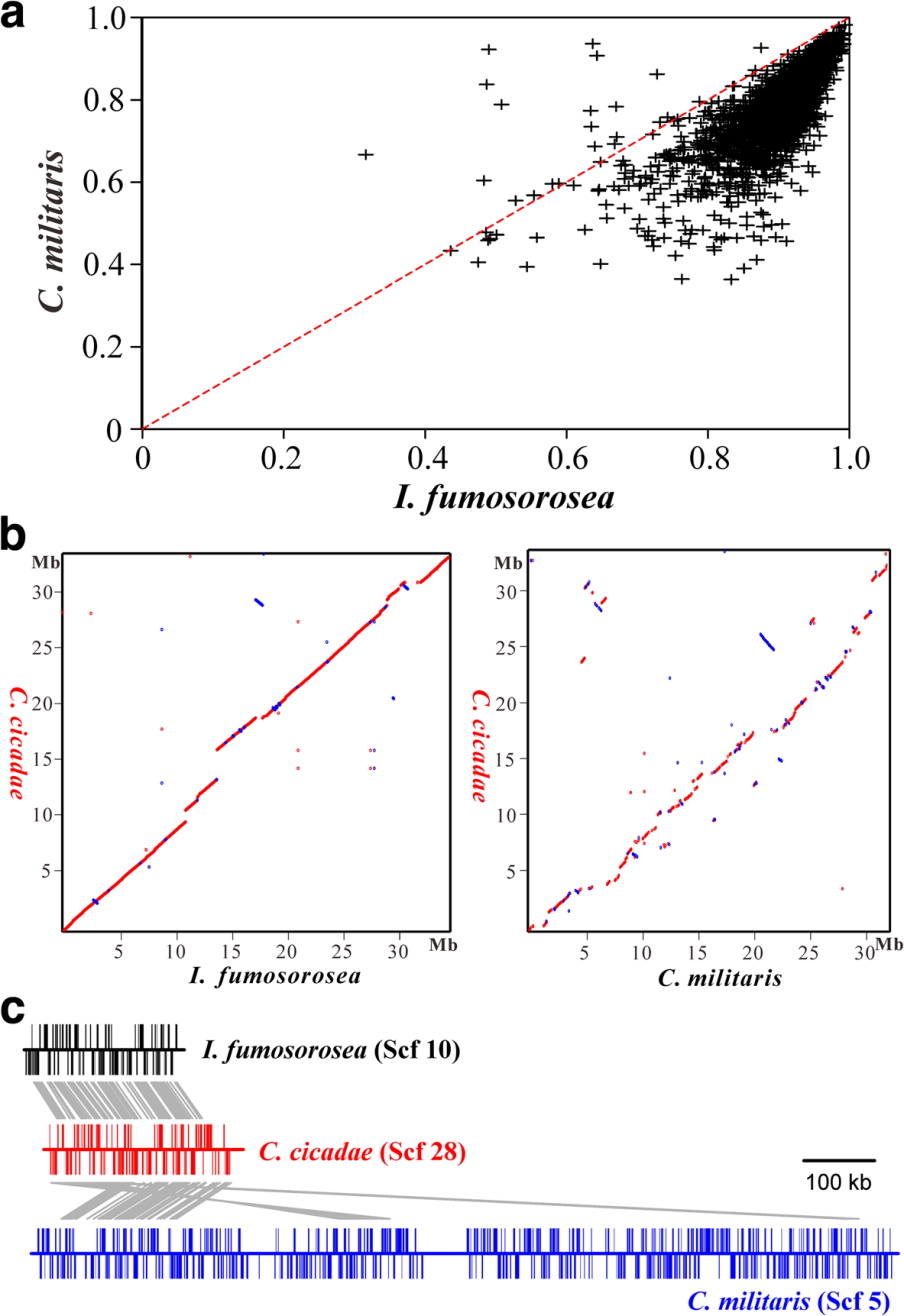
Genome structure comparison between *C. cicadae* and other closely-related fungi. **a** Scatter plots of Blast score ratio analysis of *C. cicadae* showing that the fungus is more closely related to *I. fumosorosea* than to *C. militaris*. **b** Dot blot analysis of *C. cicadae*, *I. fumosorosea* and *C. militaris* using ordered scaffold data. The blue spots show reverse-oriented relationships between the genomes. **c** Syntenic analysis of the representative scaffold structures between the three fungi. ISF, *I. fumosorosea*; CCAD, *C. cicadae*; CCM, *C. militaris*

To infer the phylogeny of *C. cicadae*, a maximum likelihood phylogenomic tree was generated by including other insect pathogens, plant pathogens, and mycoparasites using 47 single-copy and conserved orthologous protein sequences. Consistent with a previous analysis [27], we observed that the three families of *Cordyceps* species could be well separated from each other and confirmed that *C. cicadae* belongs to the Cordycipitaceae family. In addition, the phylogeny revealed that *C. cicadae* is more closely related to the *Isaria* genus than to the *Cordyceps* lineage (Additional file 1: Fig. S2). The whole genome Blast score ratio analysis also indicated that the proteins encoded by *C. cicadae* have higher similarities to those in *I. fumosorosea* than to those in *C. militaris* (Fig. 2a). In addition, the pairwise comparison analysis based on oriented scaffolds demonstrated that the genome structure of *C. cicadae* is highly syntenic with that of *I. fumosorosea* whereas the fragmented, transverse, and/or reverse-oriented relationships are observed between the genomes of *C. cicadae* and *C. militaris* (Fig. 2b and c). *C. cicadae* and *I. fumosorosea* are therefore closely related to each other.

### Divergent structure and function of mating-type loci

**Fig. 3:**
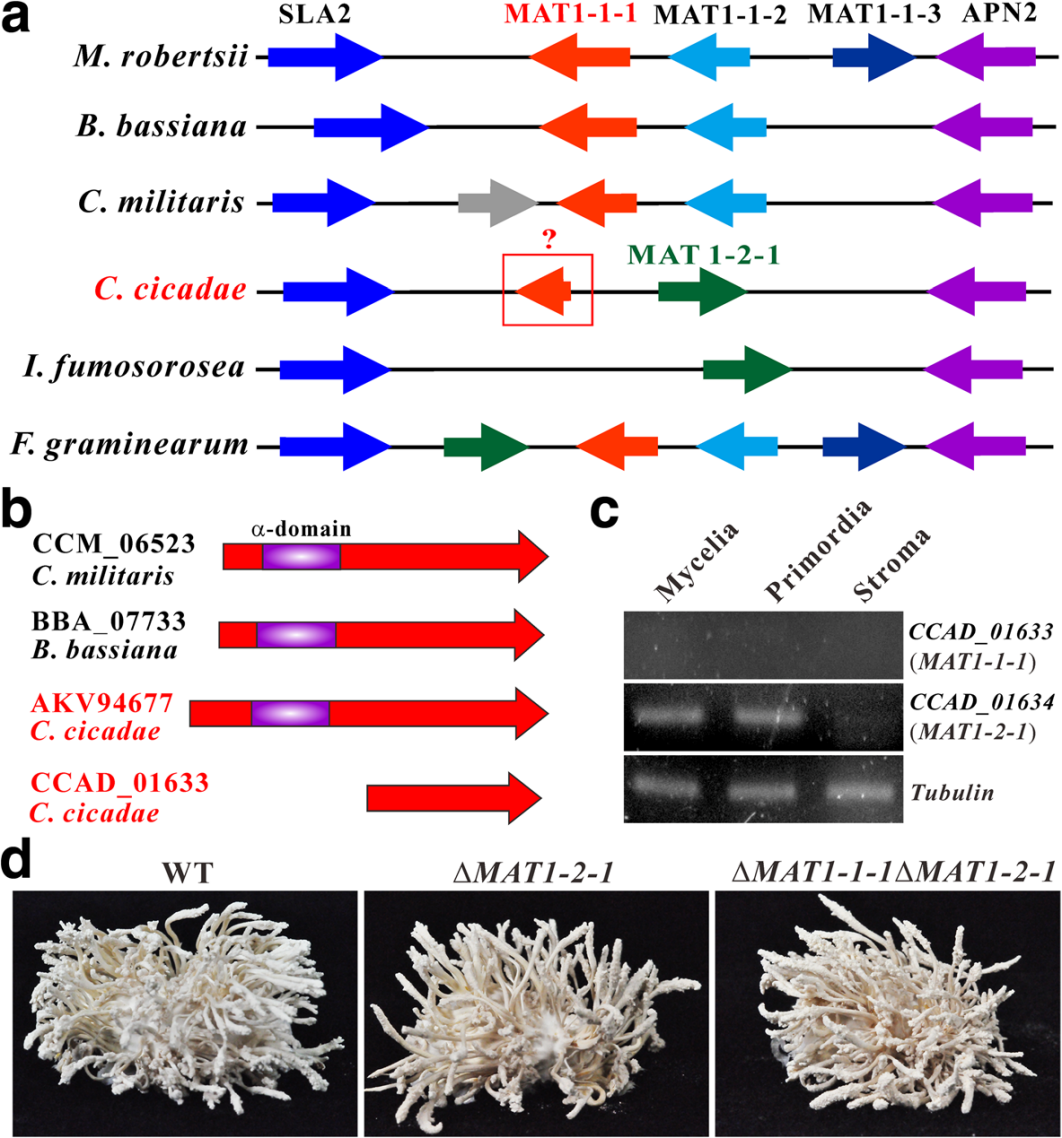
Structure, gene expression and loss-of-function analyses of the mating-type loci. **a** Structure comparison of the idiomorphic region between different fungi. In contrast to the structures of heterothallic or homothallic fungi, the MAT1-2 type of *C. cicadae* contains a truncated *MAT1-1-1* gene. **b** Structure comparison of the MAT1-1-1 protein between different fungi. The truncated MAT1-1-1 encoded in the MAT1-2 type of *C. cicadae* has lost the N-terminus α-box domain. The MAT1-1-1 (AKV94677) from a MAT1-1 type of *C. cicadae* has a structure similar to those of *C. militaris* and *B. bassiana*. **c** RT-PCR analysis of MAT genes expressed by *C. cicadae* at different developmental stages. The mycelia harvested from the SDB (3 dpi), and primordia (13 dpi) and stroma (23 dpi) produced on silkworm pupae were used for RNA extraction and gene expression analysis. **d** Fruiting body production by the WT and mutant strains. Conidia of the WT and mutant strains were injected in the tussah silkworm pupae for 25 days

Fungal sexual lifecycle is controlled by the MAT loci that are also called as the mini sex-chromosomes [11, 28]. Except for the presence of two opposite MAT loci in the haploid genome of *O. sinensis* (i.e., being homothallically sexual), a single MAT locus is observed in the haploid genomes of most *Cordyceps* species, i.e., being sexually heterothallic [11, 12]. To determine the potential mechanism of asexual fruiting in *C. cicadae*, the structure of MAT locus was examined. We identified a MAT1-2 type in the sequenced strain of *C. cicadae* (Fig. 3a). However, an additional gene (CCAD_01633, encoding a protein of 226 aa) was also identified in the MAT1-2 locus that shows similarities to the MAT1-1-1 (CCM_06523, 456 aa; 52% identity) of *C. militaris* and MAT1-1-1 (BBA_07733, 456 aa; 51% identity) of *B. bassiana*. Further analysis indicated the lack of the conserved α-domain of HMG (high mobility group) box in CCAD_01633 (Fig. 3b). Thus, a truncated type of MAT1-1-1 is present in the MAT1-2 locus of *C. cicadae*.

To examine the function of MAT1-2-1 and truncated MAT1-1-1 in controlling asexual fruiting in *C. cicadae*, we first examined the gene expression profiles during fungal developments. Consistent with the transcription profile of *MAT1-2-1* in *C. militaris* [15], *MAT1-2-1* of *C. cicadae* was transcribed during fungal growth in a liquid culture and early fruiting-body development (i.e., the primordium formation stage) but down-regulated during the fruiting-body maturation stage (i.e., stromata formation stage). Nevertheless, the truncated *MAT1-1-1*-like gene was not expressed by the fungus under the examined growth conditions (Fig. 3c). To further determine the MAT genes’ effect on fungal fruiting, single and joint gene deletions were performed by homologous replacement. We found that deletion of either single *MAT1-2-1* or both *MAT1-2-1/MAT1-1-1* had no effect on the fruiting-body formation of *C. cicadae* on caterpillar pupae (Fig. 3d). Thus, asexual fruiting of *C. cicadae* is not regulated by the MAT locus.

### Conservation and divergence of the gene clusters involved in secondary metabolisms

**Fig. 4:**
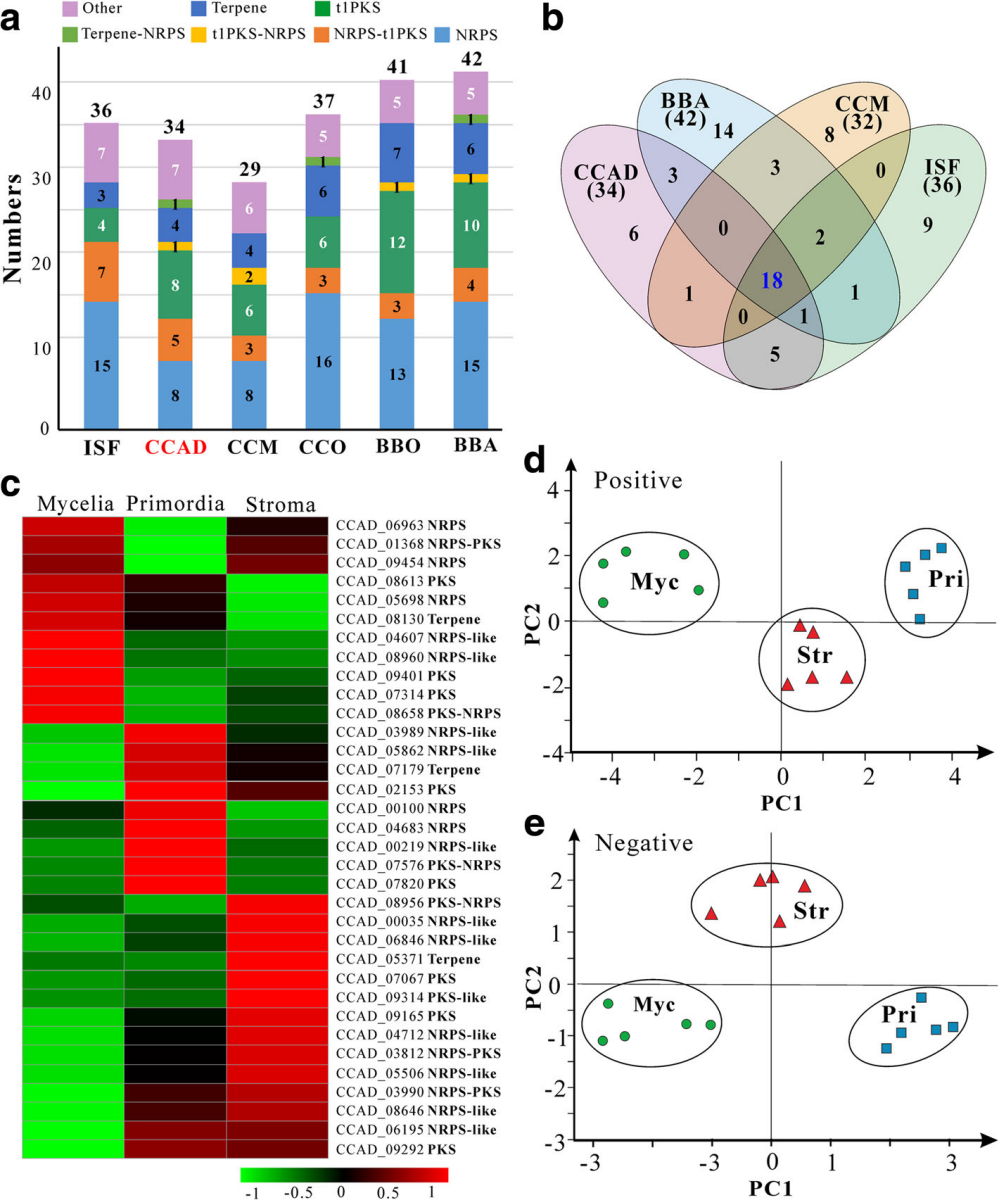
Fungal secondary metabolisms. **a** Comparison of the core genes involved in secondary metabolisms between *C. cicadae* (CCAD) and other closely-related fungi. ISF, *I. fumosorosea*; CCM, *C. militaris*; CCO, *C. confragosa* (anamorph: *Lecanicillium lecanii*); BBO, *B. brongniartii*; BBA, *C. bassiana*. **b** Venn diagram analysis of the secondary metabolic gene clusters between *C. cicadae* and other fungi. **c** Differential expression of the core secondary metabolic genes in *C. cicadae* at different developmental stages. PCA plotting based on metabolomic data obtained from the positive (**d**) and negative ion mode (**e**) of LC-MS analysis. There were five independent repeats for each sample. Myc, mycelia grown in SDB for seven days; Pri, primordia harvested from the mycosed tussah silkworm pupae 13 days post injection; Str, stroma harvested from the mycosed silkworm pupae 22 days post injection

With the obtained genome information of *C. cicadae*, we identified the gene clusters putatively involved in secondary metabolisms, including the 34 core enzymes of non-ribosomal peptide synthetase (NRPS), polyketide synthase (PKS), NRPS-PKS hybrid, and terpene synthase. We found that while *C. cicadae* encodes more gene clusters than *C. militaris* (29) it encodes fewer gene clusters than *I. fumosorosea* (36) and other selected fungi (Fig. 4a). For example, similar to *C. militaris*, fewer NRPS clusters (8 in total) are encoded in *C. cicadae* than those (average 14) in other fungi. Conservation analysis of these gene clusters revealed the presence of 18 clusters being conserved in four closely-related fungal species while six clusters are species-specific to *C. cicadae* (Fig. 4b). Consistent with their conserved genome structures (Fig. 2), *C. cicadae* shares more conserved gene clusters with *I. fumosorosea* than with other fungi. RNA-seq analysis indicated that these core enzyme genes were differentially transcribed by the fungus under different growth conditions or developmental stages. Overall, relative to the growth in Sabouraud dextrose broth (SDB), more genes were up-regulated when the fungus was grown on the silkworm pupae, especially during the maturation of fruiting bodies (Fig. 4c).

**Fig. 5:**
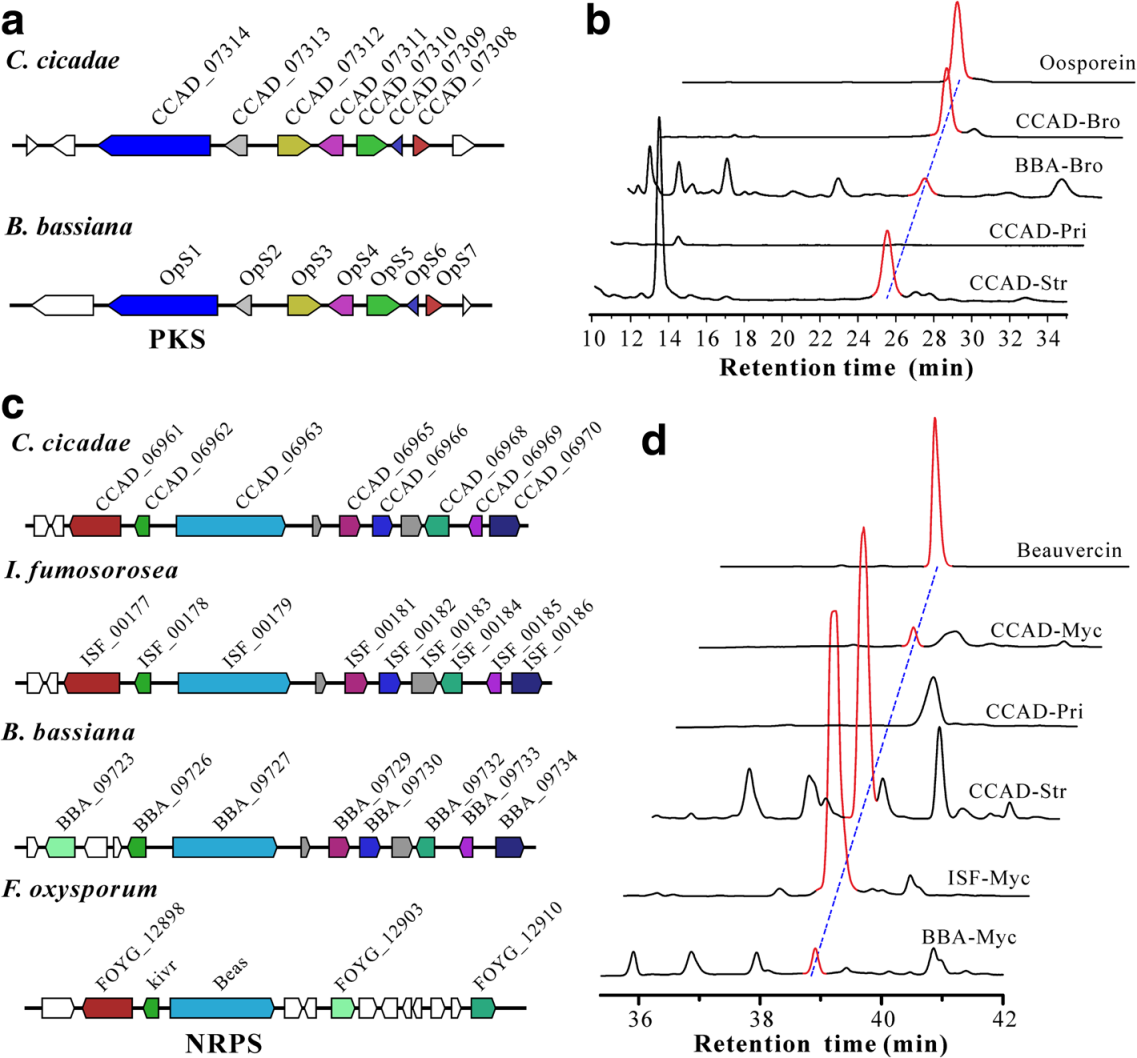
Conservation and metabolite production analysis of the gene clusters involved in biosynthesis of oosporein and beauvericin in *C. cicadae*. **a** Schematic map of the oosporein biosynthetic gene cluster in *C. cicadae* and *B. bassiana*. **b** HPLC verification of oosporein (red peak) production in *C. cicadae* and *B. bassiana*. **c** Schematic map of the beauvericin biosynthetic gene cluster in different fungi. **d** HPLC analysis of beauvericin (red peak) production in different fungi. CCAD, *C. cicadae*; BBA*, B. bassiana*; ISF, *I. fumosorosea*. Myc, mycelia harvested from SDB broth; Pri, primordia; Str, stroma

Considering that *C. cicadae* is being consumed as a medicinal/healthy fungus, we performed phylogenetic and enzyme modulation analysis of PKSs and NRPSs with the counterparts that produce human toxicogenic/carcinogenic toxins in other fungi. Phylogeny analysis based on the ketoacyl synthase domain of PKSs indicated the groupings of some PKSs encoded by *C. cicadae* with those involved in human mycotoxin-producing enzymes. However, comparative modulation analysis revealed the divergent nature of the corresponding PKSs (Additional file 1: Fig. S3). For example, CCAD_09292 clusters with the fumonisin-producing PKS but the structures of these two enzymes are different from each other. Similar analysis was performed for the NRPSs of *C. cicadae* by using the retrieved adenylation domain sequences. The results confirmed the absence of HC-toxin, gliotoxin, and enniatin-producing gene clusters in the genome of *C. cicadae* (Additional file 1: Fig. S4). On the other hand, we found that a PKS gene cluster of *C. cicadae* is highly conserved with the cluster involved in the biosynthesis of bibenzoquonine oosporein in *B. bassiana* [29] (Fig. 5a). Based on our previous protocols [29], we performed oosporein extraction and high-performance liquid chromatography (HPLC) analysis of *C. cicadae* for verification. The results confirmed that, similar to *B. bassiana*, *C. cicadae* could produce oosporein in both the liquid culture and fruiting bodies (Fig. 5b)*.* Despite the close relationship between *C. cicadae* and *I. fumosorosea* (Additional file 1: Fig. S2), the latter does not have an oosporein-biosynthetic gene cluster. A conserved NRPS cluster is also highly conserved in *C. cicadae*, *I. fumosorosea*, *B. bassiana*, and the plant pathogen *Fusarium oxysporum* (Fig. 5c). This NRPS cluster of *B. bassiana* is responsible for the production of the insecticidal cyclopeptide beauvericins [30]. HPLC analysis also verified that, similar to *B. bassiana*, both *C. cicadae* and *I. fumosorosea* could produce beauvericin (Fig. 5d). Thus, both the conserved and divergent features of secondary metabolic gene clusters are observed between *C. cicadae* and other fungi.

### Metabolomic profiles

To corroborate the differential expression of secondary metabolic genes (Fig. 4c), high throughput LC-mass spectrometry (MS) metabolomic analysis was conducted by using five independent extracts isolated from liquid mycelia, primordia and stroma formed on insect pupae. Interestingly, principal component analysis based on the LC-MS data obtained from both the positive and negative ion modes could well separate the samples into three groups in association with fungal growth conditions or developmental stages (Fig. 4d). The results indicate that fungal metabolisms (including secondary metabolisms) are tightly linked with fungal developments. Consistent with the above analysis, both oosporein and beauvericins were detected in the metabolome data (Additional file 2). In addition, the adenosine analog, *N* ^6^-(2-hydroxyethyl)-adenosine, a Ca^2+^ antagonist in mammalian cells and possessing sedative activity [31], was also identified in the LC-MS analysis of *C. cicadae*. Relative to the growth in SDB, different species of phospholipids, amino acids (e.g., arginine, threonine, glutamine and saccharopine), and organic acids (e.g., oxalic acid, fumaric acid, gluconic acid and linoleic acid) were found to be accumulated in the primordial and stromata samples (Additional file 2; Additional file 1: Fig. S5A and S5B). The metabolites previously unreported from *C. cicadae* were also identified in our metabolomic analysis (Additional file 2; Additional file 1: Fig. S5C). For example, cyclodipeptide cordysinin A, first identified in the caterpillar fungus *C. sinensis* [32], was detected in our metabolomic data. Additional compounds first identified in non-insect pathogens were also detected, including the antibiotic fumimycin first reported in *Aspergillus fumisynnematus* [33], lichenicolin A reported in a lichenicolous fungus [34], alkaloid sinensine B from the basidiomycete *Ganoderma sinense* [35], and cycloheximide acid A from a bacterium [36].

### Transcriptional profiles associated with fungal developments

We also performed high throughput RNA-seq transcriptomic analyses of *C. cicadae* grown in SDB or on silkworm pupae. Some stage-specifically expressed genes were observed but most genes (7851) were transcribed at several developmental stages (Additional file 1: Fig. S6A). At least, six transcriptional patterns associated with fungal development were evident (Additional file 1: Fig. S6B). For example, relative to the growth in artificial medium, ca. 3000 genes were up-regulated and 2500 genes were down-regulated when the fungus was grown on silkworm pupae. Otherwise, 1377 genes were specifically up-regulated, and 806 genes down-regulated in the fungus during the maturation of fruiting bodies (Additional file 1: Fig. S6B). Then, we performed gene ontology (GO) enrichment analysis of the genes that were up-regulated >2-fold with individual *P* value of *t-*test less than 0.05 between samples [37]. Different GO terms were identified with a *P* value <0.05 and FDR (false discovery rate) < 0.05 (Additional file 3). For example, relative to the mycelial sample obtained from SDB, the GO term “secondary metabolic process” was enriched 2.5-fold in primordia and 3.3-fold in stroma, which is consistent with the up-regulations of the core secondary metabolic genes (Fig. 4c). Consistent with the metabolomic data of oxalic acid (OS) accumulation in fruiting bodies (Additional file 1: Fig. S5A), the core genes involved in OS biosynthesis were found to be highly activated during fungal fruiting. For example, relative to the growth in SDB, the putative oxalate decarboxylase (CCAD_04067) [38] was up-regulated 7- and 6-fold in primordia and stroma, respectively. In addition, we found that the putative oxaloacetate acetylhydrolase (CCAD_00875) was up-regulated 137-fold in primordia and 147-fold in stroma. Considering that OS is a risk factor for the formation of calcium-oxalate kidney stones [39], OS quantification analysis was performed. Consistently, relative to the SDB mycelial samples (1.66 ± 1.04 mg/g), OS content was significantly (*P =* 0.01) increased in the fruiting-bodies (5.89 ± 0.33 mg/g) of *C. cicadae*.

We established that conidial spores instead of ascospores are produced by the asexual fruiting bodies of *C. cicadae* (Fig. 1A). The expression of the genes putatively involved in mating and meiosis was examined, and the data revealed that most of these genes were not up-regulated in *C. cicadae* during asexual fruiting (Additional file 4). Consistent with the reverse transcription PCR (RT-PCR) analysis (Fig. 3C), the truncated *MAT1-1-1* was not transcribed in the fungus whereas *MAT1-2-1* was not up-regulated during fungal fruiting. We also found that a putative alpha-factor pheromone gene (CCAD_03382), which is similar to the homolog CPP1 (50% identity) identified in *C. militaris* [15], was not transcribed in *C. cicadae*. A yeast IME1-like positive regulator of meiosis (CCAD_07081, 41% identity) [40] was expressed at similar levels by the fungus during different growth stages. The protein CCAD_04741, which is similar to the yeast SPO11 (22% identity) required for the formation of double-strand breaks and the initiation of meiotic recombination [41], was not transcribed by the fungus during the maturation of stroma. On the other hand, relative to the growth in SDB, most genes involved in conidiation were up-regulated during fungal fruiting. For example, the *Aspergillus*-like conidiophore development regulator AbaA (AN0422 vs. CCAD_06931, 40% identity) and the conidium wall factor hydrophobin RodA (AN8803 vs. CCAD_03400, 38% identity) were up-regulated 15- and 736-fold in stroma, respectively (Additional file 4). Overall, the transcriptome data support the non-mating and non-meiosis but conidiation processes of *C. cicadae* asexual fruiting.

## Discussion

Like other organisms, fungi reproduce sexually or asexually to transmit genes to the next generations [42]. We previously found that the haploid isolate of *C. militaris* could produce fruiting bodies in the laboratory with few conidia formed on the mature stroma [43], and this capacity could be lost during successive maintenance of the cultures [44]. In this study, we established that the fruiting bodies of *C. cicadae* are asexually formed in the field or could be induced in the laboratory. Unlike the same sex mating or mating-type switch in yeasts [42], the sexual structures such as perithecia, asci, and ascospores are not produced on the fruiting bodies of *C. cicadae* but the production of conidial spores. These structures are somehow similar to the synnemata produced by *C. bassiana* [3] or *I. japonica* [45]. Asexual fruiting (also called as monokaryotic fruiting) has also been observed in the basidiomycete mushrooms such as *Schizophylloum commune* [46] and *Agrocybe aegerita* [47]. However, in contrast to *C. cicadae*, the sexual structure basidia could still be formed but beard only two instead of four basidiospores during the asexual cycle of *A. aegerita*. The evolution and developmental control of asexual fruiting in *C. cicadae* remain elusive. It cannot be precluded that sexual reproduction of *C. cicadae* does not occur at all in the fields. The nymphs and pupae of cicadae are soil dwelling. Thus, similar to the “summit disease syndrome” of insects killed by some fungal species [48], the production of conidia on the protruded fruiting bodies of *C. cicadae* may have been selected to benefit the dispersal of fungal spores to initiate the next infection cycle.

The teleomorph of some *Isaria* species has been verified to be *Cordyceps* spp., for example, *C. takaomontana* for *I. tenuipes* and *C. memorabilis* for *I. farinosa* [1]. Comparative analysis of the MAT loci among different fungi indicated that *C. cicadae* MAT1-2 locus contains a truncated type of *MAT1-1-1* without the conserved α-box domain. Genome survey of the *C. cicadae* BA-001 strain sequenced by another group (GenBank accession: AEIW00000000) confirmed the similar presence of the truncated *MAT1-1-1* gene in the MAT1-2 locus, implying that this gene is not a pseudogene. The HMG-box domain of MAT1-1-1 mediates DNA binding and functions as a transcription factor to activate the mating-type specific transcription of pheromone and pheromone receptor genes [49]. The loss of α-box domain suggests that this truncated *MAT1-1-1* could be non-functional for gene activations. The deletion of α-box domain in the truncated *MAT1-1-1* has also been observed in the conifer pathogens *Grosmannia* spp. [50] and the beetle-associated fungi *Leptographium procerum* and *L. profanum* [51], suggesting a general pattern of MAT gene truncation. In contrast to our observation (Fig. 3C), the expression of the truncated *MAT1-1-1* was detected in *G. clavigera* during fungal vegetative growth on a solid medium [50]. The biological function(s) of the truncated *MAT1-1-1* in fungal sexuality and/or developmental regulation remains to be determined.

Comparative genome structure analysis revealed a high level of non-syntenic relationships between *C. cicadae* and *C. militaris*. Since *C. militaris* can readily perform sexual reproduction to increase genetic recombination [11], this finding further supports that the lifecycle of *C. cicadae* is largely asexual and clonal in nature. An additional support to the asexual lifecycle of *C. cicadae* comes from gene deletion studies that the MAT locus is not required for fruiting (Fig. 3D). Moreover, our transcriptomic data indicate that most of the genes putatively involved in mating and meiosis are not up-regulated during fruiting-body formation (Additional file 4). The mechanisms of asexual fruiting in *C. cicadae* remain to be further investigated.

Insect bioassays confirmed that, in contrast to the caterpillar-specific fungus *C. militaris*, *C. cicadae* could infect and kill non-cicada insect hosts. Comparative genomic analysis revealed that the protein families encoded in *C. cicadae* have the typical features of entomopathogenic fungi, including the expansion of serine proteases and chitinases to effectively target the protein- and chitin-rich insect hosts [2, 26]. Consistent with the trajectory of protein family expansions in *Metarhizium* species to diverge from a specialist ancestor to generalist species [13], *C. cicadae* encodes more GPCRs, effector-like SSCPs, and secondary metabolic gene clusters than *C. militaris* that may contribute to the former for recognition, immune evasion, and infection of a wider range of insect hosts. Appressorium induction assays indicated that the specialist *C. militaris* did not form appressoria on the hind wings of *Tenebrio* and failed to kill beetle larvae (Additional file 1: Fig. S1). However, the homolog of a benzoquinone oxidoreductase (BBA_01593), characterized in *B. bassiana* to detoxify benzoquinone-containing defensive secretions in the tenebrionid beetles [52], is present in *C. militaris* (CCM_06005, 85% identity) and as well as in *C. cicadae* (CCAD_01949, 86% identity), *I. fumosorosea* (ISF_00350, 79% identity), and other insect pathogens. Thus, this enzyme may not particularly contribute to the detoxification of insect protective benzoquinone secretions. Similar to *B. bassiana*, *C. cicadae* but not *C. militaris* can produce beauvericins and oosporein. Both types of metabolites have non-selective insecticidal activities [29, 30], which may additionally contribute to the adaptation of *C. cicadae* to a wide host range.

Like other TCM herbs, the safety of consuming *Cordyceps* is still concerned [24]. In Asian countries, *C. cicadae* is one of the widely used *Cordyceps* species with renopretective activity [17]. However, potential adverse side effects of consuming this fungus are poorly understood. Our genome data reveal an array of secondary metabolic genes clusters encoded in *C. cicadae*, and comparative analysis suggests that no carcinogenic mycotoxins are likely to be produced by the fungus. However, the confirmation of oosporein production in *C. cicadae* indicates that this fungus could potentially cause avian gout or mortality in chickens and birds [53, 54]. Previous in vivo and in vitro assays also indicated that oosporein could cause cytotoxicity in the canine kidney and lead to tissue damages in mice in a dose-dependent manner by inducing oxidative stress [55]. In addition, our transcriptomic and metabolomic data indicated that the oxalic acid (OS) biosynthetic pathway was up-regulated during fungal fruiting, and OS was more highly accumulated in the fruiting-body samples when compared to the liquid cultures. OS has a strong metal-chelating property [56], and the frequent dietary intake of OS/oxalate may pose a risk for kidney stone disease [39]. Frequent consumption of *C. cicadae* fruiting bodies should therefore be limited to reduce the potential risk of calcium-oxalate kidney stone disease. Biologically, OS has been elucidated as a virulence factor of plant pathogenic fungi [38, 57], and *B. bassiana* against invertebrate hosts [58]. OS production could also increase fungal tolerance to toxic metals or ability to mobilize nutrients from minerals [56, 59]. Thus, OS accumulation in *C. cicadae* may benefit fungal colonization of insect hosts and beyond. From both the safety and biological implication points of view, the physiology and metabolism of OS in *C. cicadae* require further investigation.

Consistent with previous metabolomic analyses of *B. bassiana* [60] and *Metarhizium* species [61], metabolomic investigation of *C. cicadae* not only revealed fungal development-associated metabolic patterns but also identified the bioactive molecules that have not been reported before in *C. cicadae* (Additional file 1: Fig. S5C). In addition to the identification of oosporein for the first time, we also found the production of cordysinin A, fumimycin and lichenicolin A, which have not been reported before in *C. cicadae*. Fumimycin is a peptide deformylase inhibitor and has antibacterial activity [33]. Lichenicolin A shows antibiotic activity against gram-positive bacteria [34]. The identification of these compounds can benefit the medicinal uses of *C. cicadae*. On the other hand, some metabolites previously reported in *C. cicadae* were not detected in this study. For example, cordycecin A [20] and myriocin [62] were not identified in our metabolomic data. Consistent with a previous report [22], we also found that *C. cicadae* does not produce the adenosine analog cordycepin but *N* ^6^-(2-hydroxyethyl)-adenosine. Overall, future investigations are required to fully elucidate the chemical constituents of *C. cicadae*.

## Conclusions

In conclusion, we performed comprehensive omics analyses of the medicinal fungus *C. cicadae* in this study. The combined data reveal the uncommon biological features and genetic nature of asexual fruiting in this fungus. Genome mining and comparative analysis of core enzymes for secondary metabolisms and metabolomic inspections support to some extent the safe consumption of *C. cicadae* fruiting bodies. Future efforts are still required to fully dissect the biosynthetic potentials of this fungus for pharmaceutical explorations and safety concerns as well.

## Methods

### Fungal strain and maintenance

The *C. cicadae* strain CCAD02 was isolated from the fruiting body of a mycosed cicada sample collected from Zhejiang Province, China. Cultures of *C. cicadae* were maintained either in SDB (Difco) or on potato dextrose agar (PDA, Difco). For fruiting body induction, conidial spore suspension (2 × 10^7^ conidia/ml) was injected into the pupae (20 μl each) of Chinese Tussah silkworm (*Antheraea pernyi*) or inoculated on the artificial rice medium as described [63]. The strains of *M. robertsii* ARSEF 23, *B. bassiana* ARSEF 2860, *C. militaris* Cm 01 and *I. fumosorosea* ARSEF 2679 [64] were also used for comparative insect bioassays.

### Appressorium induction and insect bioassays

Conidial spores of each species harvested from the two-week old complete medium (CM) [65] were subjected to appressorium induction on the hind wings of the mealworm beetle *Tenebrio molitor* for 24 h. The samples were then examined under a Nikon microscopy (Eclipse Ni-U). To determine the virulence of different entomopathogenic fungi, insect bioassays were conducted against the last instar larvae of *T. molitor*. Thus, 20 insects per group were sprayed with 0.8 ml of the aqueous suspension (1 × 10^7^ conidia/ml). Each sample had three repeats and the experiments were repeated three times. Mortality was recorded every 12 h and the median lethal time (LT_50_) was calculated by Kaplan-Meier analysis [66].

### Genome sequencing, assembly and annotation

The genome of CCAD02 strain was sequenced with the Illumina HiSeq2500 system at the National Center for Gene Research, Chinese Academy of Sciences (Shanghai, China). Three DNA libraries with the fragment sizes of 200-300 bp, 700-800 bp and 5 kb were constructed for sequencing, and the obtained data were assembled with SOAPdenovo2 (version 2.23) using a *k*-mer value of 51. After filling the gaps with the program GapCloser (version 1.12), the scaffolds were then assembled with the contigs to construct the draft genome. The gene structures of *C. cicadae* were predicted with a combination of different algorithms as we described previously [67, 68]. A mapping analysis for core eukaryotic genes was performed to assess the completeness of the genome [69]. The whole project has been deposited at GenBank under the accession no. MWMN00000000.

### Phylogenomic and syntenic analysis

A set of 47 conserved and single-copy proteins used for generating robust fungal phylogeny [64, 70] was selected from *C. cicadae* and 25 other fungal species. The orthologous proteins were aligned with the program MUSCLE (ver. 3.8.31) [71], and the concatenated amino acid sequences were used to generate a maximum likelihood tree using the program TREE-PUZZLE [72]. For pairwise syntenic analysis of genome structures, the scaffolds of the paired genomes of *C. cicadae*, *C. militaris* and *I. fumosorosea* were oriented by MEGABLAST and Argo Genome Browser for dot plotting analysis [67]. Blast score ratio (BSR) analysis was conducted to compare the difference between the paired genomes [73]. The normalized pairs of BSR indices were visualized with the R program (version 3.1.3).

### Protein family classifications

All predicted gene models of *C. cicadae* were subjected to InterProScan analysis for identifying conserved protein families. To identify potential pathogenicity genes, whole genome blast analysis was conducted against the protein sequences catalogued at the Pathogen-Host Interaction database [74] and the top hits were selected with a cut-off *E* value of 1e-5. The protease families were classified by Blastp analysis against the MEROPS peptidase database (*E* < 1e-50). G-protein coupled receptors and GH families were classified by protein blast analyses against the GPCRDB (http://gpcrdb.org/), and CAZy databases (http://www.cazy.org/), respectively. To identify fungal secondary metabolite gene clusters, the genome data was analyzed with the program AntiSMASH (version 3.0.4) [75]. For phylogenetic analysis, the adenylation domain sequences of NRPS enzymes or the ketoacyl synthase-domain sequences of PKS proteins were extracted and aligned with Clustal X 2.0. The phylogenetic trees were generated with the program MEGA7 [76] with a Dayhoff model, 1000 bootstrap replications and pair-wise deletions for gaps or missing data.

### Mating-type gene express assays and gene deletions

Genome analysis indicated that the sequenced strain of *C. cicadae* is a MAT1-2 type but containing a truncated *MAT1-1-1* in the MAT locus. To determine the function of MAT genes in controlling *C. cicadae* fruiting, RT-PCR analysis of *MAT1-2-1* (CCAD_01634) and truncated *MAT1-1-1* (CCAD_01633) was performed using different primer pairs (Additional file 1: Table S5). A β-tubulin gene (CCAD_032660) was used as a reference. Null mutants of *MAT1-2-1*, and *MAT1-1-1*/*MAT1-2-1* (i.e. double deletions) were generated by homologous replacement as we described previously [15]. In brief, the 5′- and 3′-flanking sequences of the target gene were amplified with the corresponding primers (Additional file 1: Table S5) using the genomic DNA as a template and the PhataTM Super Fidelity DNA Polymerase (Vazyme, Piscataway, NJ, USA). The amplified products were cloned into the corresponding enzyme-restriction sites of the binary vector pDHt-Bar (conferring resistance against ammonium glufosinate) for *Agrobacterium*-mediated transformation of the WT strain. Drug-resistant transformants were selected and verified by PCR analysis. Fruiting-body induction assays were conducted using the silkworm pupae as described above.

### Metabolomic analysis

To determine the chemical constituents of *C. cicadae* grown at different developmental stages, the conidia harvested from two-week’s old PDA plates were inoculated into SDB and incubated in a rotatory shaker at 220 rpm for 7 days at 25 °C. The mycelia were harvested and washed twice with sterile water. The nascent (primordia) and mature (stroma) fruiting bodies induced on silkworm pupae were harvested. Each sample had five independent replicates. The mycelia and fruiting bodies were lyophilized at −80 °C using a freeze-dryer (Labconco Corporation), and the homogenized samples (30 mg each) were extracted with 1 ml of methanol (with the addition of 2 mg/l Fmoc-Glycine as an internal standard) by ultrasonication for 0.5 h and kept at 4 °C for 12 h. The samples were filtered through a 0.22 μm syringe filter before analysis. LC-MS analysis was performed using an Agilent 6210 TOF/Q-TOF LC-Mass Spectrometer System equipped with an electrospray ionization (ESI) source. The samples were analyzed in ESI positive and negative ion modes [77]. Aliquots of 5 μl sample were injected for analysis, and the MS data were collected between m/z 50 and 1000 Da. The raw data were subjected to MassHunter software for molecular feature extraction and then processed with the program MetaboAnalyst (ver. 3.0) for normalization [78]. PCA analysis was performed to determine the optimal separation of samples. Heatmap analysis of the identified compounds was performed using the program MultiExperiment Viewer (ver. 4.9.0).

### HPLC verification of oosporein and beauvericin productions

To verify the productions of oosporein and beauvericin, *C. cicadae* and *B. bassiana* and or *I. fumosorosea* were used for parallel incubations, metabolite extractions and HPLC analysis. To determine oosporein production, the spores of *C. cicadae* (strain CCAD02) and *B. bassiana* (strain ARSEF 2860) were inoculated in SDB and incubated in a rotatory shaker at 220 rpm for 7 days at 25 °C. The mycelia were harvested for oosporein extraction and HPLC analysis using a LC-20 AD HPLC system (Shimadzu Scientific Instruments) equipped with a Athena C18 reverse phase column (4.6 × 250 mm, 5 μm) as we previously described [29]. For beauvericin detection, the cultures of *C. cicadae*, *B. bassiana* and *I. fumosorosea* were extracted with methanol and analyzed with the same HPLC system. The mobile phase consisted of phase A (water) and phase B (acetonitrile). The gradient program was 0-40 min, 20-100% B; 40-55 min, 100% B; 55-56 min, 100%-20% B; 56-60 min 20% B. The flow rate was set at 1 ml/min and the elution was monitored at 210 nm [30].

### RNA-seq transcriptome analysis

To corroborate the metabolomic analysis and determine fungal development-associated transcriptional profiles, high throughput RNA-seq transcriptomic analysis was performed. Thus, the samples obtained in parallel for metabolomic analysis, i.e., the SDB mycelia, primordia and stroma harvested from insect pupae, were used for RNA extractions with the QIAGEN RNeasy Plant Mini Kit. Three independent replicates were prepared for each sample. cDNA libraries were constructed and sequenced with the Illumina HiSeq2500 system. The reads with only one copy detected or those could be mapped to different transcripts were excluded in further analysis. Other tags were mapped to the genome or annotated genes if they possessed no more than one nucleotide mismatch. The read data have been deposited at NCBI with an accession no. SRP100593. The level of gene transcription was converted to fragment per kb of exon model per million mapped reads (FPKM) for expressional comparison between samples. Differentially expressed genes with more than 2-fold change and a cut-off *P* value of less than 0.05 were subjected to GO enrichment analysis with the program DAVID (ver. 6.7) [37]. The GO terms were considered to be significantly enriched with the cutoff statistics of *P* < 0.05 and FDR < 0.05.

### Quantification of oxalic acid

The content of oxalic acid was quantitatively determined by displacement reaction with tribromoarsenazo and zirconium adjusted from the previous protocol [79]. In brief, 30 mg (dry weight) of the mycelial and fruiting body samples obtained above were homogenized and extracted with 2 ml of water by ultrasonication at 40 kHz for 30 min. The supernatants were collected by centrifugation and the pigments were removed using activated carbon. The supernatants were then individually added with 0.5 ml of 5 mM tribromoarsenazo, 0.55 ml of 0.5 g/l zirconium and 1 M HCl to a total reaction volume of 4 ml for 30 min. The optical density of each repeat was measured at 650 nm. The content of oxalic acid in each sample was determined by reference to the standard curve generated with the anhydrous oxalic acid (99%, Aladdin).

## Additional files

Additional file 1: Contains supplemental results and figure legends to the supplemental **Figs. S1** to **S6** as well as the supplemental **Tables S1.** to **Table S5**. **Figure S1**, Spore differential induction and insect bioassays. **Figure S2**, Phylogenetic relationship of *C. cicadae*. **Figure S3.** and **S4.** Phylogenetic and modular analysis of PKSs (Fig. S3) and NRPSs (Fig. S4) encoded in *C. cicadae* in comparison with those involved in the production of human mycotoxins. **Figure S5**, Metabolomic analysis of *C. cicadae*. **Figure S6**, Transcriptomic profiling of *C. cicadae* at different developmental stages. **Table S1**, Comparative analysis of putative protease genes between *C. cicadae* and other insect pathogens. **Table S2**, Comparative analysis of carbohydrate-degrading enzymes between *C. cicadae* and other insect pathogens. **Table S3**, Comparative analysis of putative lipase genes between *C. cicadae* and other insect pathogens. **Table S4**, Comparative analysis of putative bacterial-like protein toxins between *C. cicadae* and other insect pathogens. **Table S5**, Primers used in this study. (DOCX 2852 kb)

Additional file 2: Relative quantification of the metabolites produced by *C. cicadae* at different developmental stages. (XLSX 31 kb)

Additional file 3: GO analysis of the genes up-regulated between different samples of *C. cicadae.* (XLSX 51 kb)

Additional file 4: Expression of putative mating and meiosis process-related genes by *C. cicadae* at different growth stages. (XLSX 13 kb)

## Abbreviations

GH: Glycoside hydrolase
GPCR: G-protein coupled receptor
HPLC: High-performance liquid chromatography
MAT: Mating type
NRPS: Non-ribosomal peptide synthetase
OS: Oxalic acid
PKS: Polyketide synthase
SSCPs: Small secreted cysteine-rich proteins
TCMs: Traditional Chinese medicines
