# Supplementary material for: Omics data reveal the unusual asexual-fruiting nature and secondary metabolic potentials of the medicinal fungus Cordyceps cicadae
Source: BMC Genomics. 2017 Aug 30;18:668. doi: 10.1186/s12864-017-4060-4 (PMC5577849; doi:10.1186/s12864-017-4060-4)
Supplement: Supplementary file 1 — Contains supplemental results and figure legends to the supplemental Figs. S1 to S6 as well as the supplemental Tables S1. to Table S5. Figure S1, Spore differential induction and insect bioassays. Figure S2, Phylogenetic relationship of C. cicadae. Figure S3. and S4. Phylogenetic and modular analysis of PKSs (Fig. S3) and NRPSs (Fig. S4) encoded in C. cicadae in comparison with those involved in the production of human mycotoxins. Figure S5, Metabolomic analysis of C. cicadae. Figure S6, Transcriptomic profiling of C. cicadae at different developmental stages. Table S1, Comparative analysis of putative protease genes between C. cicadae and other insect pathogens. Table S2, Comparative analysis of carbohydrate-degrading enzymes between C. cicadae and other insect pathogens. Table S3, Comparative analysis of putative lipase genes between C. cicadae and other insect pathogens. Table S4, Comparative analysis of putative bacterial-like protein toxins between C. cicadae and other insect pathogens. Table S5, Primers used in this study. (DOCX 2852 kb) [file 12864_2017_4060_MOESM1_ESM.docx]

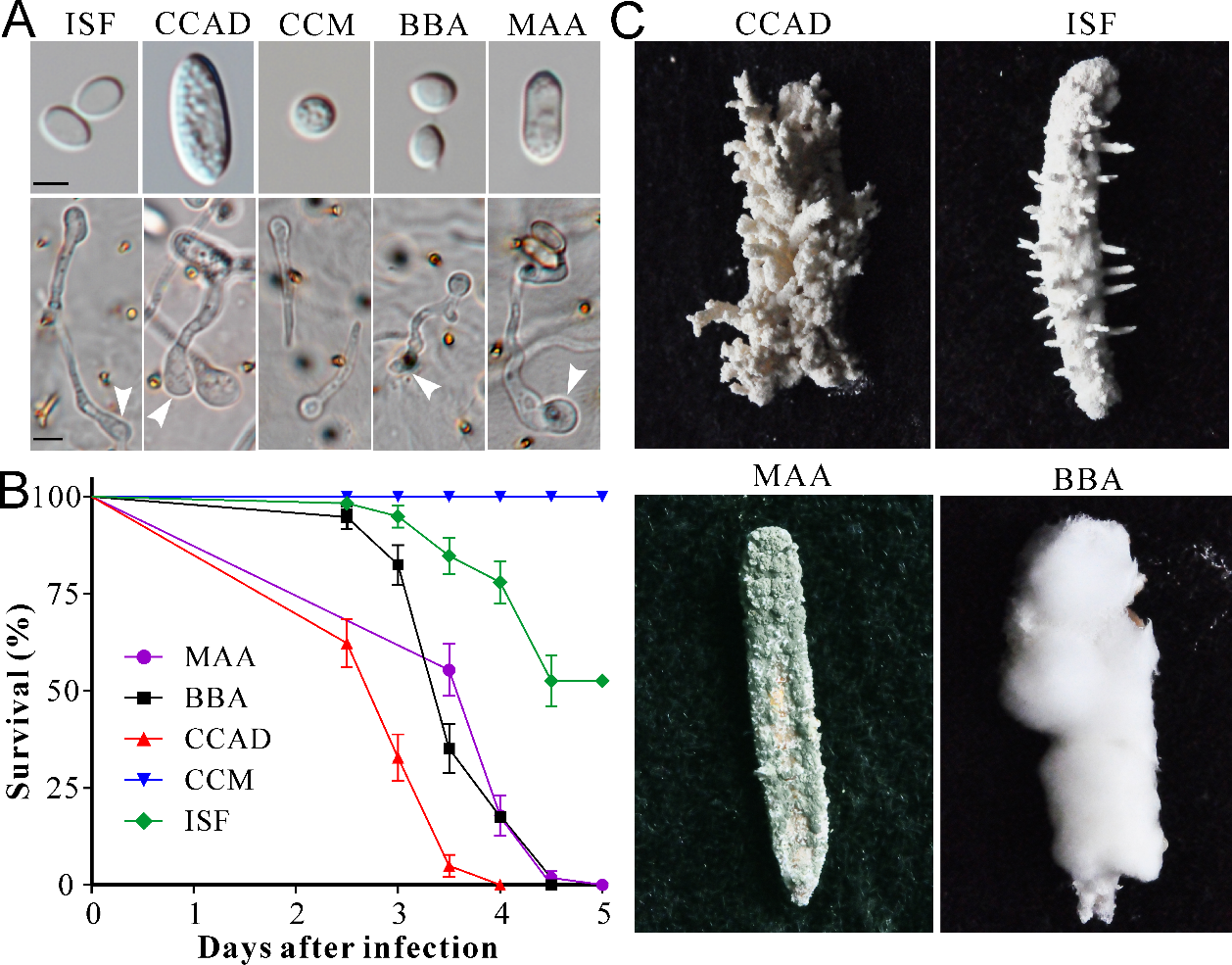


**Figure S1** Infection structure induction and insect bioassays. **a** Induction of appressorium formation on the hind wings of *Tenebrio molitor* for 18 hrs. Bar, 5 μm. **b** Survival of the mealworm larvae after topical application of the conidial suspensions (1×10^7^ conidia/ml) of different fungi. **c** Insect cadavers killed and mycosed by different fungi. Dead insects were kept on moisturized filter paper for 10 days. CCAD, *C. cicadae*; CCM, *C. militaris*; ISF, *I. fumusorosea*; MAA, *M. robertsii*; BBA*, B. bassiana.*


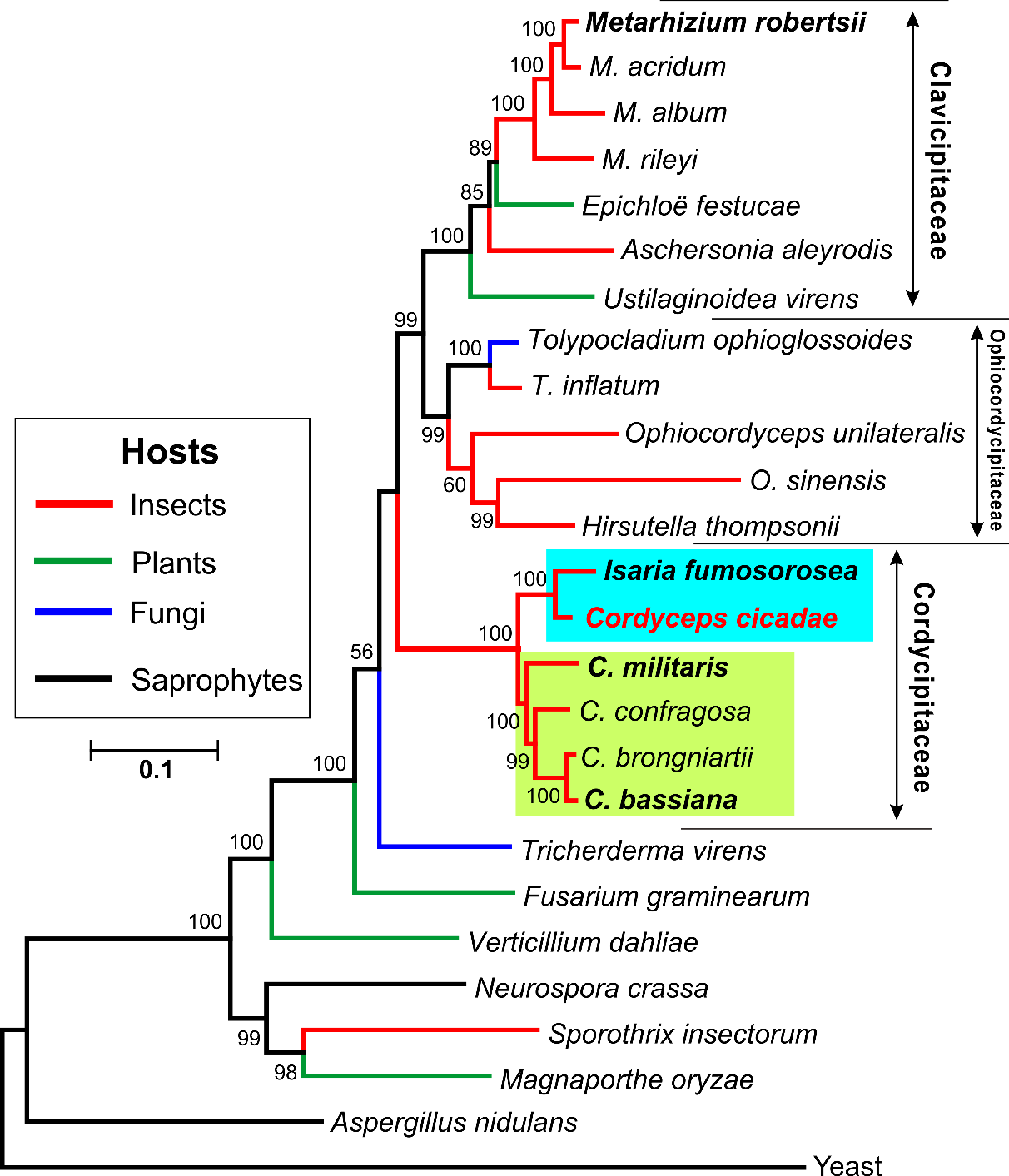


**Figure S2** Phylogenetic relationship of *C. cicadae*. The well-supported maximum likelihood tree was generated using the concatenated protein sequences of 35,242 amino acid residues. Branches labeled in different colors show fungal species association with different hosts.


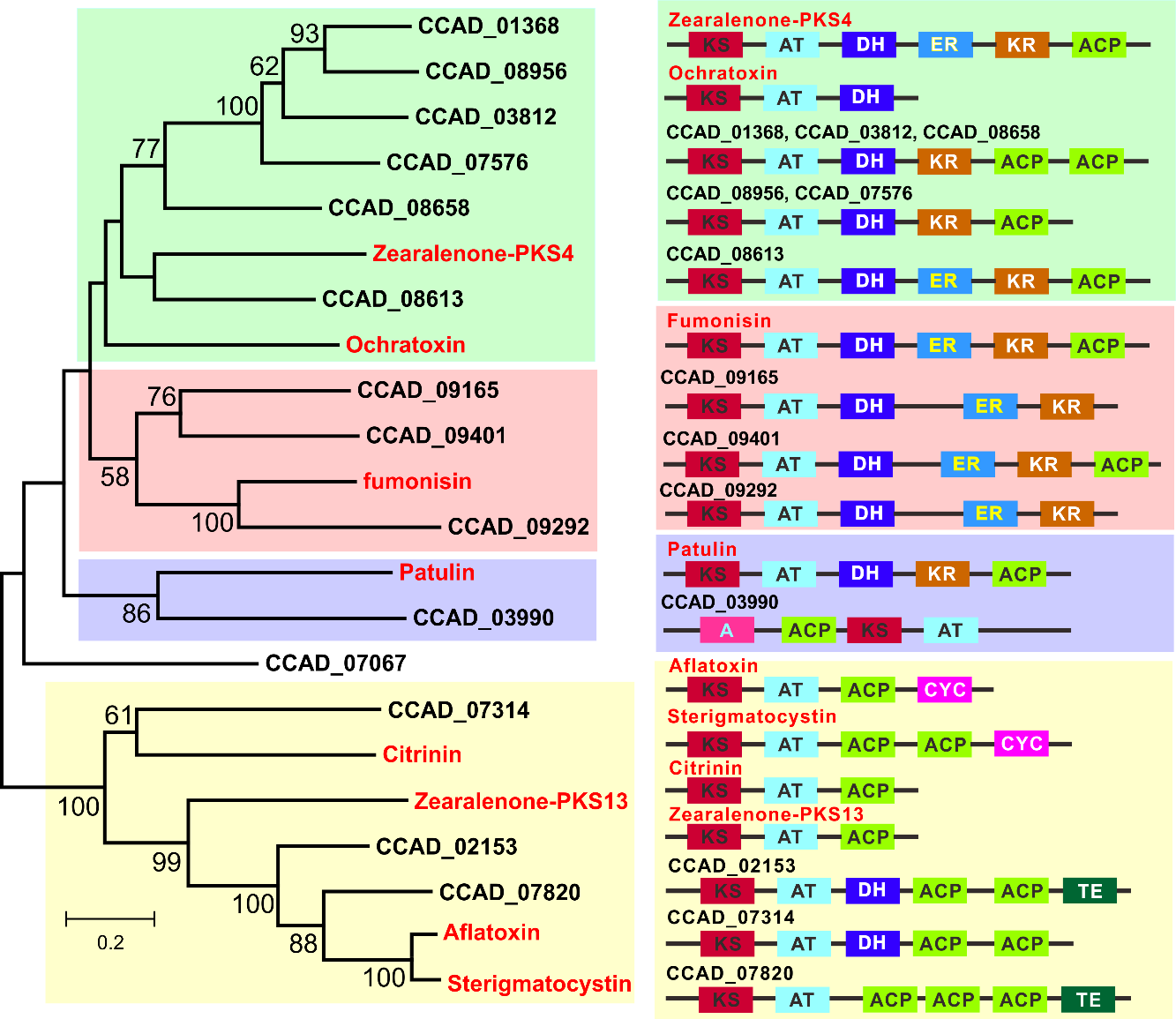


**Figure S3** Phylogenetic and modular analysis of PKSs encoded in *C. cicadae* in comparison to those involved in the production of human mycotoxins. A neighbor-joining tree was generated using the ketoacyl CoA synthase domain sequences extracted from each protein. KS, ketoacyl CoA synthase domain; AT, acyltransferase domain; ACP, acyl carrier protein domain; CYC, cyclase domain; DH, dehydratase domain; ER, enoyl reductase domain; KR, ketoreductase domain; MT, methyltransferase domain; TE, thioesterase domain.


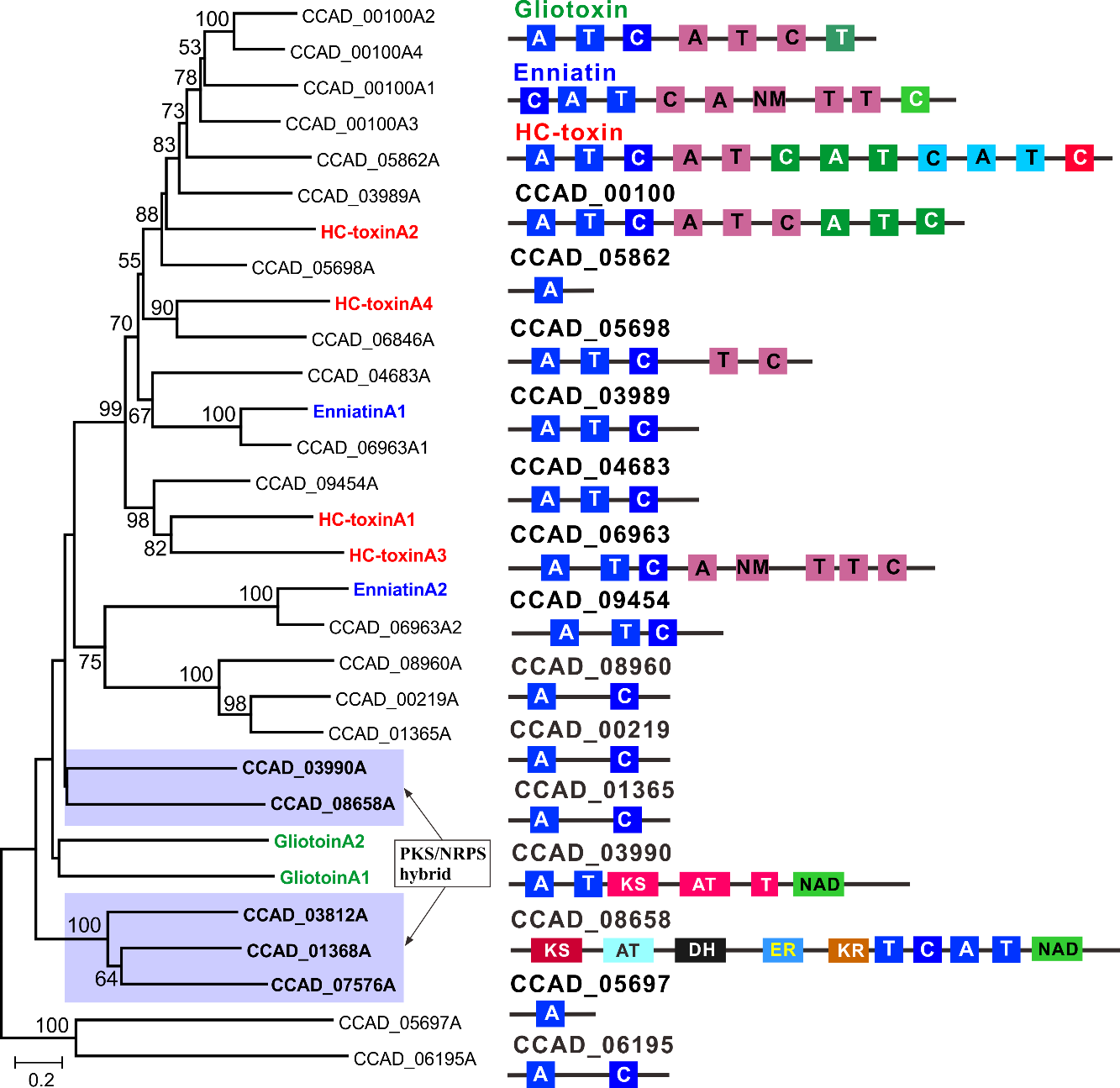


**Figure S4** Phylogenetic and modular analysis of NRPSs encoded in *C. cicadae* in comparison to those involved in the production of human mycotoxins. A neighbor-joining tree showing the relationships of adenylation domain sequences and modulation comparison of *C. cicadae* NRPSs with those involved in production of mycotoxins. A, adenylation domain; T, peptidyl carrier domain; C, condensation domain. NM, N-methyl transferase domain; KS, ketoacyl CoA synthase domain; AT, acyltransferase domain; ER, enoyl reductase domain; KR, ketoreductase domain; NAD, NAD binding.


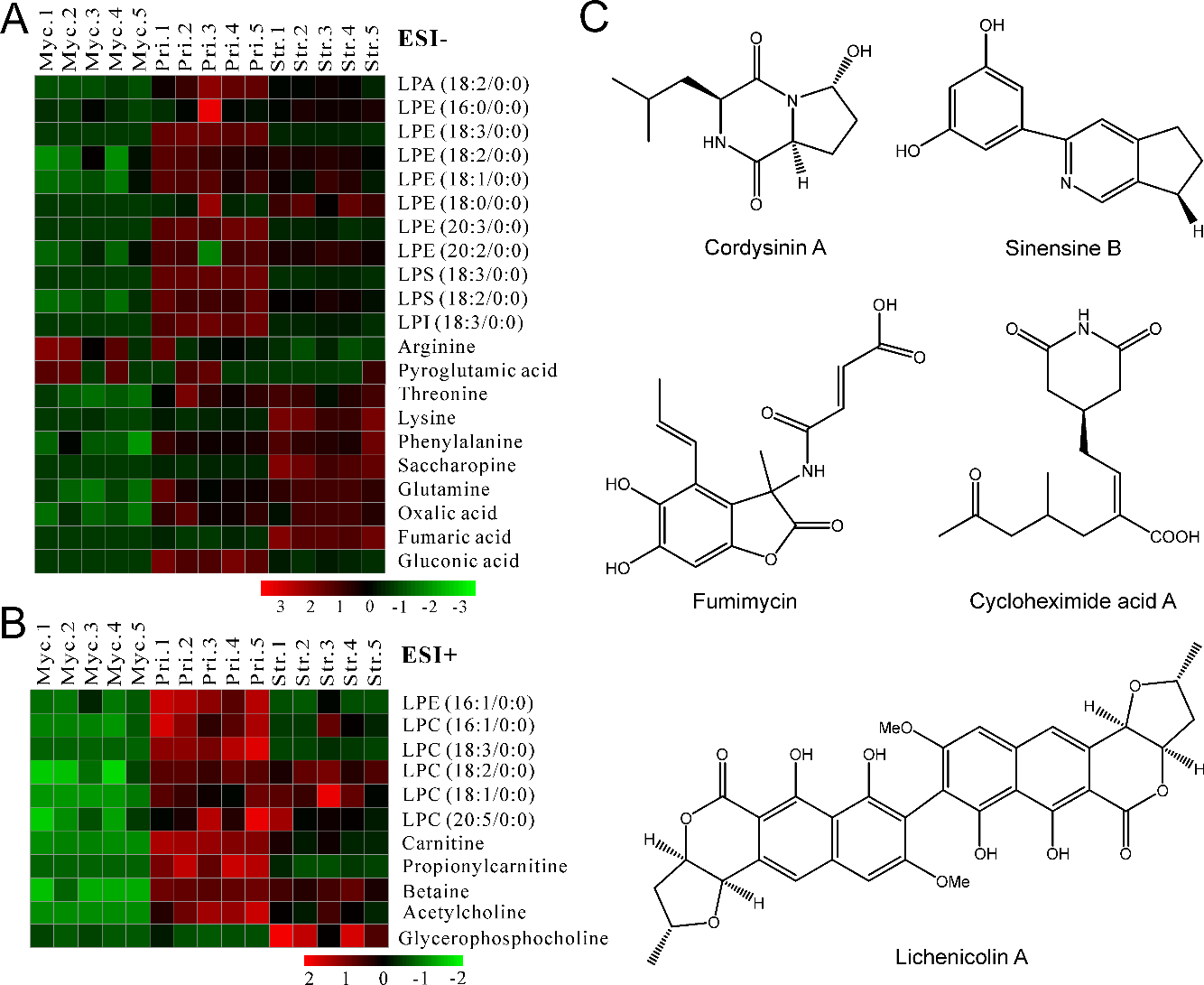


**Figure S5** Metabolomic analysis of *C. cicadae*. **a** Heatmap analysis of selected compounds detected in negative ion mode of LC-MS. **b** Heatmap analysis of selected compounds detected in positive ion mode of LC-MS. RNA samples were extracted from the mycelia (Myc) grown in SDB for four days, primordia (Pri) and stroma (Str) developed on the mycosed silkworm pupae 13 days or 22 days post injection, and used for RNA-seq analysis. There are five independent repeats for each sample. **c** Chemical structures of the selected metabolites produced by *C. cicadae*.


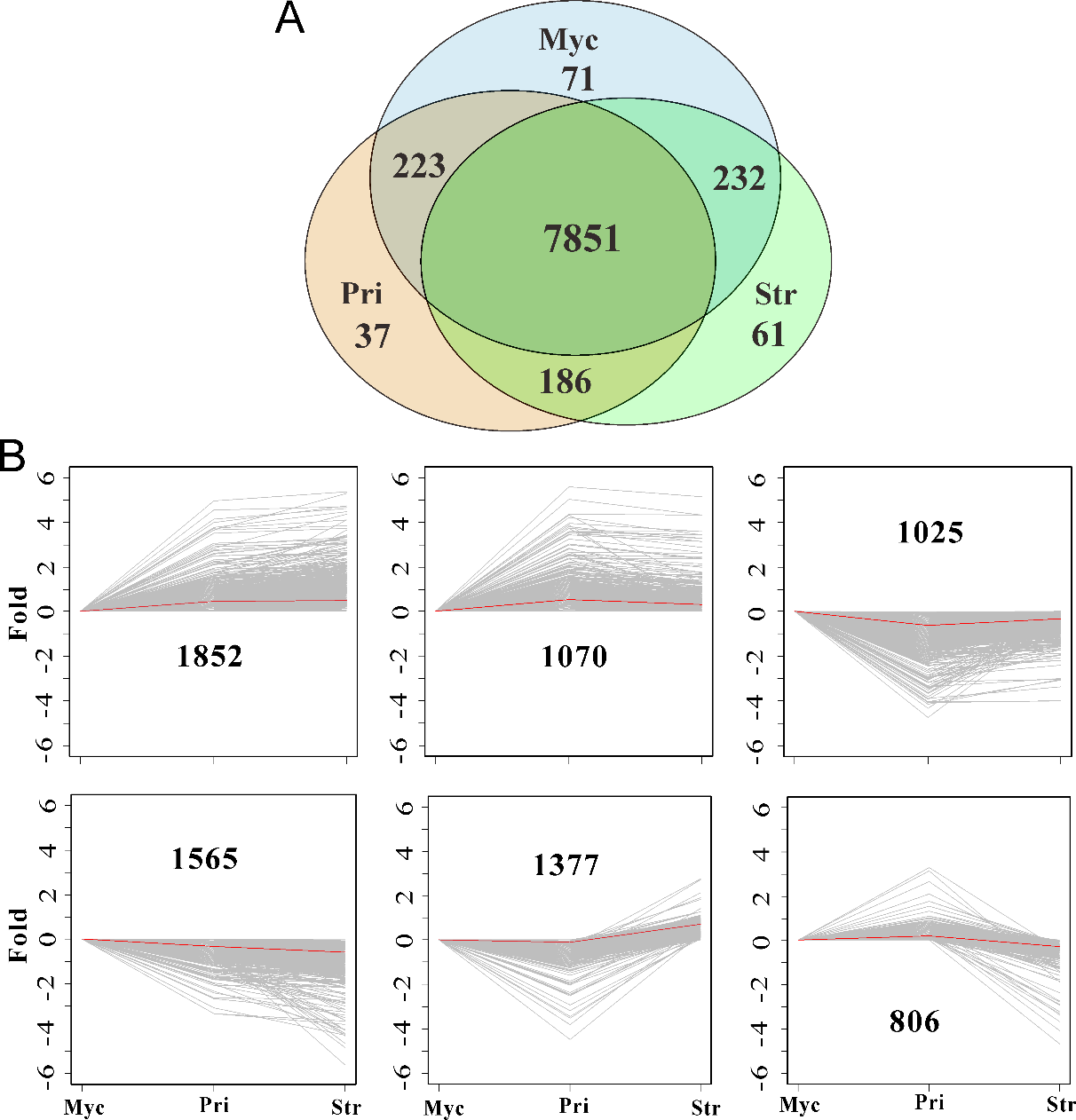


**Figure S6** Transcriptomic profiling of *C. cicadae* at different developmental stages. **a** Venn diagram analysis of genes expressed by *C. cicadae* at different developmental stages. **b** Transcriptional profiling of gene expressions by *C. cicadae* at different developmental stages. The genes with common expression patters were grouped together with the number shown in each panel. RNA samples were extracted from the mycelia (Myc) grown in SDB for four days, primordia (Pri) and stroma (Str) developed on the mycosed silkworm pupae 13 days or 22 days post injection, and used for RNA-seq analysis.

**Table S1** Comparative analysis of putative protease genes between *C. cicadae* and other insect pathogens.

| **Family** | **CCAD** | **ISF** | **CCM** | **BBA** | **MAA** |
| --- | --- | --- | --- | --- | --- |
| A01 | 19 | 20 | 21 | 21 | 22 |
| C40 | 0 | 1 | 1 | 1 | 1 |
| C44 | 0 | 0 | 0 | 0 | 0 |
| C56 | 0 | 1 | 0 | 1 | 0 |
| C69 | 1 | 0 | 1 | 0 | 1 |
| G01 | 9 | 13 | 8 | 6 | 4 |
| I09 | 0 | 1 | 1 | 2 | 0 |
| I51 | 0 | 1 | 0 | 0 | 0 |
| M06 | 1 | 0 | 0 | 0 | 1 |
| M12 | 0 | 1 | 0 | 1 | 0 |
| M14 | 4 | 3 | 4 | 3 | 2 |
| M19 | 0 | 0 | 1 | 0 | 0 |
| M20 | 2 | 2 | 2 | 2 | 2 |
| M23 | 0 | 0 | 0 | 0 | 0 |
| M28 | 5 | 6 | 5 | 4 | 5 |
| M35 | 0 | 2 | 2 | 1 | 3 |
| M36 | 0 | 0 | 1 | 0 | 1 |
| M43 | 12 | 14 | 8 | 11 | 14 |
| S01 | 8 | 11 | 7 | 13 | 28 |
| S08 | 12 | 19 | 12 | 21 | 32 |
| S09 | 8 | 13 | 9 | 9 | 10 |
| S10 | 10 | 13 | 10 | 10 | 8 |
| S12 | 8 | 7 | 4 | 11 | 6 |
| S26 | 4 | 2 | 2 | 3 | 2 |
| S28 | 3 | 5 | 4 | 4 | 3 |
| S33 | 4 | 6 | 4 | 5 | 6 |
| S53 | 15 | 15 | 11 | 11 | 7 |
| S54 | 0 | 0 | 1 | 0 | 0 |
| T02 | 0 | 0 | 0 | 0 | 1 |
| T03 | 2 | 2 | 1 | 2 | 1 |
| Total | 127 | 158 | 120 | 142 | 160 |

Fungal species: CCAD, *C. cicadae*; CCM, *C. militaris*; ISF, *I. fumusorosea*; BBA, *B. bassiana*; MAA, *M. robertsii*.

**Table S2** Comparative analysis of carbohydrate-degrading enzymes between *C. cicadae* and other insect pathogens.

| **Family** | **CCAD** | **ISF** | **CCM** | **BBA** | **MAA** |
| --- | --- | --- | --- | --- | --- |
| GH1 | 0 | 0 | 2 | 2 | 4 |
| GH10 | 0 | 0 | 0 | 1 | 0 |
| GH105 | 0 | 0 | 0 | 0 | 1 |
| GH115 | 0 | 0 | 0 | 0 | 1 |
| GH125 | 3 | 3 | 2 | 3 | 2 |
| GH13 | 6 | 8 | 6 | 6 | 7 |
| GH15 | 3 | 3 | 4 | 2 | 3 |
| GH16 | 7 | 4 | 3 | 5 | 9 |
| GH17 | 3 | 2 | 2 | 2 | 3 |
| GH18 | 16 | 18 | 20 | 20 | 21 |
| GH2 | 8 | 9 | 7 | 8 | 6 |
| GH20 | 4 | 4 | 3 | 5 | 2 |
| GH27 | 4 | 2 | 1 | 2 | 2 |
| GH28 | 1 | 0 | 0 | 0 | 0 |
| GH29 | 0 | 0 | 1 | 1 | 1 |
| GH3 | 8 | 10 | 9 | 10 | 7 |
| GH31 | 4 | 6 | 4 | 6 | 6 |
| GH32 | 1 | 1 | 1 | 1 | 1 |
| GH33 | 0 | 0 | 0 | 0 | 1 |
| GH35 | 3 | 3 | 3 | 3 | 2 |
| GH36 | 3 | 3 | 2 | 2 | 2 |
| GH37 | 2 | 2 | 2 | 2 | 2 |
| GH38 | 2 | 2 | 2 | 2 | 3 |
| GH4 | 0 | 1 | 0 | 2 | 1 |
| GH43 | 2 | 2 | 2 | 2 | 1 |
| GH47 | 8 | 8 | 7 | 7 | 8 |
| GH5 | 6 | 7 | 7 | 7 | 8 |
| GH54 | 3 | 3 | 1 | 1 | 1 |
| GH55 | 6 | 4 | 8 | 7 | 5 |
| GH63 | 1 | 1 | 1 | 1 | 1 |
| GH64 | 1 | 1 | 1 | 1 | 1 |
| GH65 | 1 | 1 | 1 | 0 | 1 |
| GH71 | 2 | 4 | 2 | 3 | 3 |
| GH72 | 6 | 6 | 6 | 7 | 6 |
| GH75 | 3 | 2 | 2 | 2 | 1 |
| GH76 | 9 | 10 | 9 | 8 | 13 |
| GH79 | 3 | 2 | 3 | 2 | 2 |
| GH81 | 1 | 1 | 1 | 1 | 1 |
| GH84 | 1 | 1 | 1 | 1 | 1 |
| GH88 | 0 | 1 | 1 | 1 | 1 |
| GH89 | 1 | 3 | 2 | 2 | 2 |
| GH92 | 3 | 4 | 4 | 4 | 5 |
| GH93 | 0 | 1 | 1 | 1 | 1 |
| GH95 | 0 | 0 | 0 | 1 | 0 |
| Total | 135 | 143 | 134 | 144 | 149 |

Fungal species: CCAD, *C. cicadae*; CCM, *C. militaris*; ISF, *I. fumusorosea*; BBA*, B. bassiana*; MAA, *M. robertsii*.

**Table S3** Comparative analysis of putative lipase genes between *C. cicadae* and other insect pathogens.

| **Family** | **CCAD** | **ISF** | **CCM** | **BBA** | **MAA** |
| --- | --- | --- | --- | --- | --- |
| abH01 | 1 | 3 | 3 | 2 | 3 |
| abH03 | 4 | 0 | 1 | 2 | 3 |
| abH04 | 4 | 3 | 3 | 2 | 6 |
| abH07 | 4 | 4 | 3 | 7 | 10 |
| abH08 | 0 | 0 | 1 | 1 | 0 |
| abH09 | 2 | 0 | 0 | 1 | 3 |
| abH13 | 1 | 0 | 0 | 1 | 1 |
| abH14 | 2 | 2 | 2 | 2 | 2 |
| abH27 | 2 | 2 | 3 | 3 | 2 |
| abH32 | 0 | 0 | 0 | 0 | 0 |
| abH34 | 8 | 7 | 7 | 7 | 5 |
| abH38 | 1 | 0 | 0 | 0 | 0 |
| Total | 29 | 21 | 23 | 28 | 35 |

Fungal species: CCAD, *C. cicadae*; CCM, *C. militaris*; ISF, *I. fumusorosea*; BBA*, B. bassiana*; MAA, *M. robertsii.*

**Table S4** Comparative analysis of putative bacterial-like protein toxins between *C. cicadae* and other insect pathogens.

| **Family** | **CCAD** | **ISF** | **CCM** | **BBA** | **MAA** |
| --- | --- | --- | --- | --- | --- |
| Heat-labile enterotoxin, A chain | 7 | 5 | 1 | 13 | 9 |
| Bordetella pertussis toxin A | 2 | 0 | 0 | 1 | 0 |
| Delta endotoxin, N-terminal | 4 | 2 | 1 | 8 | 0 |
| Zeta toxin | 2 | 1 | 1 | 3 | 1 |
| Killer toxin, Kp4/SMK-like | 0 | 4 | 3 | 0 | 5 |
| Insecticidal toxin complex/plasmid viulence protein | 0 | 0 | 0 | 0 | 1 |
| Insecticide toxin TcdB middle/N-terminal | 1 | 0 | 0 | 0 | 0 |
| clostridium neurotoxin,translocation | 0 | 0 | 0 | 1 | 0 |
| Total | 16 | 12 | 6 | 26 | 16 |

CCAD, *C. cicadae*; CCM, *C. militaris*; ISF, *I. fumusorosea*; BBA*, B. bassiana*; MAA, *M. robertsii.*

**Table S5** Primers used in this study.

| **Genes** | **Primers** | **Primer sequence** | **Note** |
| --- | --- | --- | --- |
| *MAT1-2-1* | 121-UF | TTCCTGCAGCCCGGGGGATCCCGGCCAACTTTTCATCATCTG | Forward and reverse primers for application of the upstream of MAT121 gene for deletion. |
|  | 121-UR | TCATCTTCTGTCGACGGATCCGATCACTCCGCCTTGTTGATCA |  |
|  | 121-DF | ACCGAGATCTGATGAACTAGTCAAAAGGCTACCATGTGCCAG | Forward and reverse primers for application of the downstream of MAT121 gene for deletion. |
|  | 121-DR | GGCGGCCGCTCTAGAACTAGTCGGTAAATGAGAGGCGTAGCG |  |
| *MAT1-2-1/ MAT1-1-1* | 111-UF | TTCCTGCAGCCCGGGGGATCCGGGGGCATAATGAAGATGTGC | Forward and reverse primers for application of the upstream of MAT121/MAT1-1-1 gene for deletion. |
|  | 111-UR | TCATCTTCTGTCGACGGATCCTTCTCGAGGGTGCTGTTTCTATATC |  |
|  | 121-DF | ACCGAGATCTGATGAACTAGTCAAAAGGCTACCATGTGCCAG | Forward and reverse primers for application of the downstream of MAT121/MAT1-1-1 gene for deletion. |
|  | 121-DR | GGCGGCCGCTCTAGAACTAGTCGGTAAATGAGAGGCGTAGCG |  |
| *MAT1-1-1* | 111-RT-F | CTCCATCTACAGCCCGAGTC | For RT-PCR analysis of *MAT1-1-1* |
|  | 111-RT-R | CCGATAGCATGCCCATAGAT |  |
| *MAT1-2-1* | 121-RT-F | AGCGAGCCACATTTGTCAAA | For RT-PCR analysis of *MAT1-2-1* |
|  | 121-RT-R | TGGGTGTCGTTCAAGGAGAG |  |
| *Tubulin* | Tubulin-F | TTCCCTCGTCTGCACTTCTT | Used as a RT-PCR reference |
|  | Tubulin-R | CCTCCTTCATGGCAACCTTA |  |
